# Global genetic diversity and evolutionary patterns among *Potato leafroll virus* populations

**DOI:** 10.3389/fmicb.2022.1022016

**Published:** 2022-09-26

**Authors:** Tahir Farooq, Muhammad Dilshad Hussain, Muhammad Taimoor Shakeel, Hasan Riaz, Ummara Waheed, Maria Siddique, Irum Shahzadi, Muhammad Naveed Aslam, Yafei Tang, Xiaoman She, Zifu He

**Affiliations:** ^1^Guangdong Academy of Agricultural Sciences, Plant Protection Research Institute and Guangdong Provincial Key Laboratory of High Technology for Plant Protection, Guangzhou, China; ^2^State Key Laboratory for Agro-Biotechnology, and Ministry of Agriculture and Rural Affairs, Key Laboratory for Pest Monitoring and Green Management, Department of Plant Pathology, China Agricultural University, Beijing, China; ^3^Department of Plant Pathology, Faculty of Agriculture & Environment, The Islamia University of Bahawalpur, Bahawalpur, Pakistan; ^4^Institute of Plant Protection, Muhammad Nawaz Shareef University of Agriculture, Multan, Pakistan; ^5^Institute of Plant Breeding and Biotechnology, Muhammad Nawaz Shareef University of Agriculture, Multan, Pakistan; ^6^Department of Environmental Sciences, COMSATS University Islamabad, Abbottabad, Pakistan; ^7^Department of Biotechnology, COMSATS University Islamabad, Abbottabad, Pakistan

**Keywords:** *Potato leafroll virus*, Polerovirus, phylogenetics, recombination, mutation, evolution, selection pressure, intrinsically disordered proteins

## Abstract

Potato leafroll virus (PLRV) is a widespread and one of the most damaging viral pathogens causing significant quantitative and qualitative losses in potato worldwide. The current knowledge of the geographical distribution, standing genetic diversity and the evolutionary patterns existing among global PLRV populations is limited. Here, we employed several bioinformatics tools and comprehensively analyzed the diversity, genomic variability, and the dynamics of key evolutionary factors governing the global spread of this viral pathogen. To date, a total of 84 full-genomic sequences of PLRV isolates have been reported from 22 countries with most genomes documented from Kenya. Among all PLRV-encoded major proteins, RTD and P0 displayed the highest level of nucleotide variability. The highest percentage of mutations were associated with RTD (38.81%) and P1 (31.66%) in the coding sequences. We detected a total of 10 significantly supported recombination events while the most frequently detected ones were associated with PLRV genome sequences reported from Kenya. Notably, the distribution patterns of recombination breakpoints across different genomic regions of PLRV isolates remained variable. Further analysis revealed that with exception of a few positively selected codons, a major part of the PLRV genome is evolving under strong purifying selection. Protein disorder prediction analysis revealed that CP-RTD had the highest percentage (48%) of disordered amino acids and the majority (27%) of disordered residues were positioned at the C-terminus. These findings will extend our current knowledge of the PLRV geographical prevalence, genetic diversity, and evolutionary factors that are presumably shaping the global spread and successful adaptation of PLRV as a destructive potato pathogen to geographically isolated regions of the world.

## Introduction

**Figure 1:**
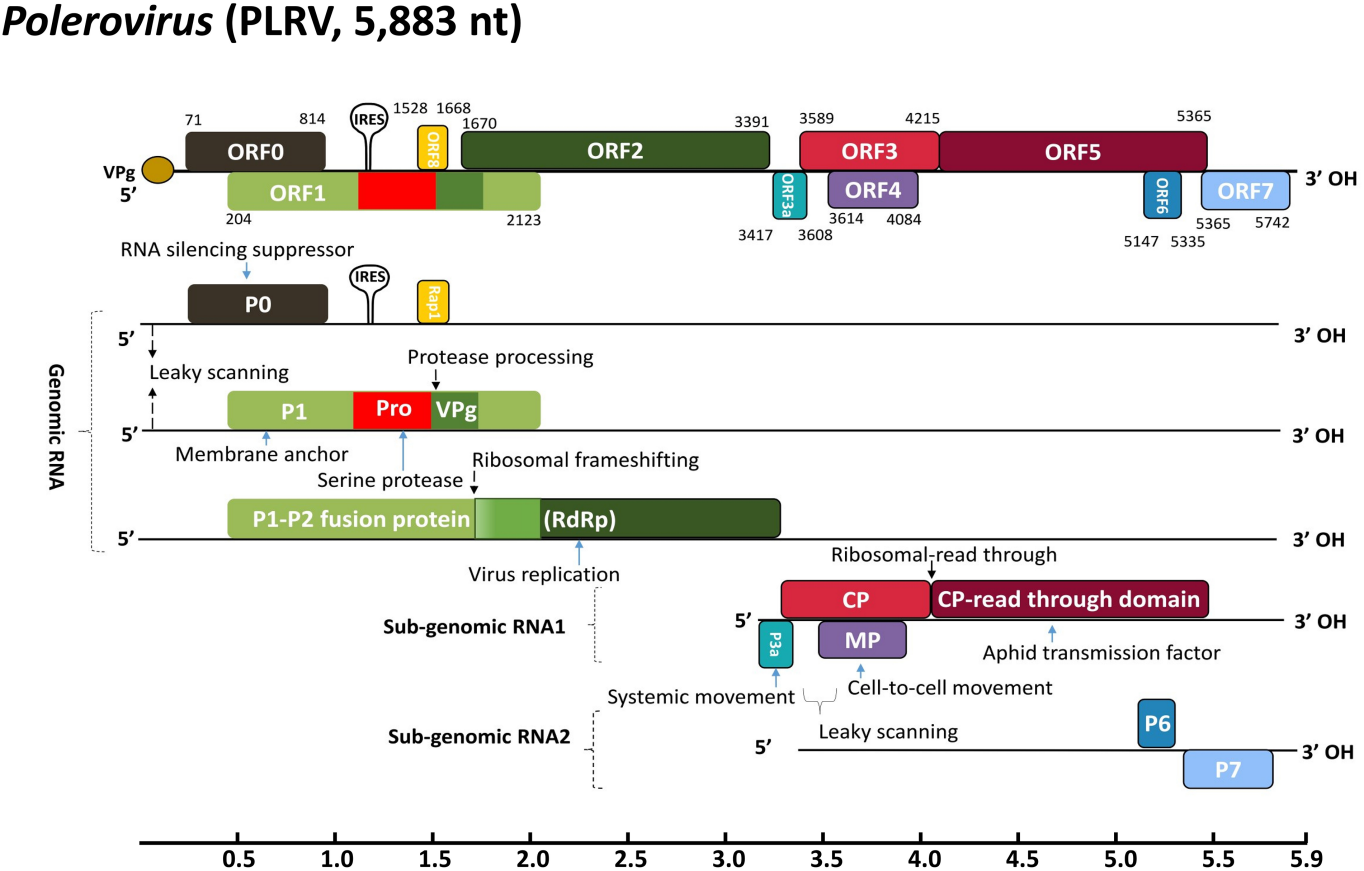
Schematic representation of PLRV genomic organization and strategies for gene expression. The ribosomal frameshifts and read-through strategies are indicated. The types and positions of different ORFs along with corresponding translated proteins are represented by colored boxes while solid lines denote the non-coding regions. The abbreviations denote VPg, viral genome-linked protein; IRES, internal ribosomal entry site; Rap1, replication-associated protein 1; CP, coat protein; MP, movement protein; RdRp, RNA-dependent RNA polymerase. The genome annotation is based on the full genome sequence of PLRV (GenBank accession: D13954.1).

The genetic diversity facilitates virus evolution and adaptation to new environments and regulates acute viral infections to species from all of life’s domains being parasitized. Factually, massive diseases around the globe suspected to be caused by viruses have been documented for millennia (Wasik and Turner, 2013; Kumar et al., 2017; Gelbart et al., 2020; Rubio et al., 2020; Pascall et al., 2021; Harvey and Holmes, 2022). Potato leafroll Polerovirus (PLRV), from the genus *Polerovirus* and the family *Solemoviridae*, consists of a 5.3–5.7 kb long positive sense (+) monopartite single-stranded RNA [(+)ssRNA] genome with virus protein genome-linked (VPg) cap bounded at the 5′ end and an OH group at the 3′ end without poly(A) tail or tRNA-like organization (LaTourrette et al., 2021; Walker et al., 2021). Typically, the PLRV genome contains 6–7 overlapping open reading frames (ORFs), which are organized into genomic and sub-genomic RNAs (Figure 1; Krueger et al., 2013; Sõmera et al., 2015; Barrios Barón et al., 2017). The RNA-dependent RNA polymerase (RdRp), expressed *via* ribosomal frameshifting, is a translational fusion of ORF1 that encodes P1 and ORF2, which encodes P2. Additionally, a Rap1 translation initiates through a peculiar internal ribosome entry site (IRES) around 1,500 nt downstream of the 5′ end of the gRNA. From sub-genomic RNA 1 (sgRNA1), a capsid protein (CP), involved in virion formation, vector transmission, and virus movement, is encoded from ORF3 (Kaplan et al., 2007; Smirnova et al., 2015). Subsequently, ORF3 extends and ribosomes incorporate one amino acid and continue to translate ORF5 into a CP-read through domain (CP-RTD) as a fusion protein of the translational fusion of ORF3 and ORF5, which is involved in vector transmission and virus movement (Peter et al., 2008; Boissinot et al., 2014; Xu et al., 2018). Furthermore, leaky scanning of sgRNA1 results in the expression of P3a from ORF3a, reported for virus long-distance movement, and P4 from ORF4, a phloem restricted or cell-to-cell movement protein. In addition, PLRV encodes P6 (ORF6) and P7 (ORF7) from the sub-genomic RNA 2 (sgRNA2; Figure 1). The P7 protein plays the most important role in the enhancement of aphid fecundity (Patton et al., 2020). Interestingly, the characteristic feature of poleroviruses is the presence of ORF0 that encodes P0 protein, significantly involved in the viral suppressing of RNA silencing (VSR). The P0 with CP and VPg also contributes to vector specificity (Pazhouhandeh et al., 2006; Baumberger et al., 2007; Csorba et al., 2010; Patton et al., 2020). There are more than 50 viruses that infect potatoes (Kreuze et al., 2020); among them, PLRV is a major pathogen of potato (*Solanum tuberosum*) and transmitted by aphids (*Myzus persicae*) being dreadfully responsible for annual production losses of more than 20 million tones globally (Kumari et al., 2020; Patton et al., 2020). It is ranked the second most prevalent pathogen in Asia, Africa, Europe, North America, and South America, threatening the sustainable food production system (CABI, 2010). In most cases, viral interaction may occur in the host as the result of mixed viral infection leading to disease severe epidemics. PLRV in association with potato potyvirus Y (PVY) causes additional losses to the marketable potato production industry (Palukaitis, 2012; Valkonen, 2015; Okeyo, 2017; Byarugaba et al., 2020).

The frequent emergence of new viral diseases is primarily due to the ability of the viruses to rapidly evolve. Mostly, viruses retain genomic flexibility in order to adapt to various hosts and vectors (Rantalainen et al., 2011; Garcia-Ruiz, 2018; Nigam and Garcia-Ruiz, 2020; Rubio et al., 2020). Virus evolution and host adaptation are merely determined through genetic diversity among viral populations (Obenauer et al., 2006; Moury and Simon, 2011; Gelbart et al., 2020; Nigam and Garcia-Ruiz, 2020; Rubio et al., 2020; Farooq et al., 2021; LaTourrette et al., 2021). This viral evolutionary process, which is mediated by RNA recombination and preferential accumulation of mutations in certain portions of the genome, leads to the emergence of new viral strains with exceptional characteristics (Obenauer et al., 2006; Moury and Simon, 2011; Dombrovsky et al., 2013; Ibaba et al., 2017; Nigam and Garcia-Ruiz, 2020; LaTourrette et al., 2021). In general, poleroviruses evolve due to the most common genetic variations, which occur in an area encoding VPg, RdRp, and CP, and in a non-coding intergenic region between ORF2 and ORF3 at 5’ UTR of the sub-genomic RNA 1 (Pagán and Holmes, 2010; Dombrovsky et al., 2013; Ndikumana et al., 2017; Kwak et al., 2018; LaTourrette et al., 2021). Previous studies have revealed that poleroviruses exhibit higher single-nucleotide polymorphisms (SNPs) on ORFs encoding P0, P1, and CP-RTD and lower SNPs between ORFs, encoding P2 through P4, with exception of a conserved region of P2 within the P1–P2 (Huang et al., 2005; Delfosse et al., 2021; LaTourrette et al., 2021).

Since the PLRV genome comprises overlapping ORFs, mutations may affect various proteins to mediate virus evolution. However, comprehensive knowledge regarding the geographical distribution, standing genetic diversity, and evolutionary patterns existing among global PLRV populations is currently not available, which is crucial for designing sustainable disease management strategies. To fulfill the knowledge gap, we vigorously analyzed the recent global biodiversity, genomic variations, evolutionary endpoints, and the patterns of disordered proteins to gain further insights into the genetic complexity and molecular variability among PLRV populations. To date, with the advances in next-generation sequencing technology, a total of 84 full-length genome sequences of PLRV isolates have been reported across 22 different countries. PLRV isolates revealed a high level of genetic variability while the distribution patterns of recombination breakpoints across various genomic regions of PLRV isolates remained variable and most of its populations are evolving under strong purifying selection. These findings will expand our knowledge of PLRV geographical prevalence, genetic diversity, and evolutionary forces that are presumably governing the continual PLRV global spread and successful adaptation to different ecosystems.

## Materials and methods

### Acquisition of full-length PLRV genomic sequences

A total of 84 PLRV full-length genomic RNA sequences were retrieved from the GenBank database^1^ on 20 July 2022. Detailed information about the attributes (accession, isolate, country of origin, host, and sequence length) of these sequences is given in Supplementary Table S1. These 84 PLRV sequences were selected for the subsequent analyses based on the criteria; partial sequences (<95% coverage of the reference genome), sequences with >2.5% of unknown characters, and sequences with <90% similarity with reference genome have been excluded.

### Multiple sequence alignments (MSAs) and phylogenetic analysis

The MSAs were prepared by aligning 84 full-length genomic RNA sequences of globally reported PLRV isolates derived from the GenBank database (Supplementary Table S1), using the MUSCLE tool in the software Geneious Prime version 9.0.2. Likewise, alignments of the individual PLRV genes (*P0*, *P1*, *RdRp*, *CP*, *CP-RTD*, *MP*, and *RTD*) among corresponding genes of the globally reported PLRV isolates were performed. All alignments were manually analyzed and adjusted (when necessary) before proceeding to the subsequent analysis. The phylogenetic tree was constructed in the molecular evolutionary genetics analysis (MEGA-X) software by the maximum likelihood (ML) method with 1,000 bootstrap replicates (Kumar et al., 2018). The tree was visualized and annotated using iToL (Letunic and Bork, 2021). Finally, the distribution and matrix of pairwise identities among all PLRV isolates were determined using Sequence Demarcation Tool (SDT) v1.2 (Muhire et al., 2014).

### Estimation of nucleotide diversity and haplotype variability indices

The nucleotide diversity or *π* (represented by the average pairwise number of nucleotide differences per site) was calculated using DnaSP V.5 (Librado and Rozas, 2009). The significant differences in the average nucleotide diversity among all PLRV sequences were estimated by calculation of their 95% bootstrap confidence intervals. A 100-nt sliding window with a step size of 10 nts across the full-length sequences of PLRV was implicated to calculate *π*. Additional population genetics-related parameters including the number of haplotypes (H), haplotype diversity (H_d_), the number of polymorphic sites (S), Watterson’s theta (θ), the total number of mutations (Eta), and Tajima’s D were also estimated for PLRV genomes and individual coding sequences (*P0*, *P1*, *RdRp*, *CP*, *CP-RTD*, *MP,* and *RTD*) using DnaSP V.5 (Librado and Rozas, 2009).

### Recombination analysis

The occurrence of recombination events across 84 full-length PLRV sequences was investigated by using several methods including Rdp, SisterScan, Bootscan, Chimera, Geneconv, maximum Chi-square, and 3Seq. The recombination analysis was implemented in the recombination detection program (RDP) V.4 (Martin et al., 2015). For all methods, alignments were performed with default settings. The *p*-values less than the Bonferroni-corrected cutoff (0.05) were used to infer the statistically significant results. The signals detected by at least four methods were regarded as reliable recombination events.

### Analysis of positive and negative selection

The identification of potential positively and negatively selected sites in the coding sequences of P0, P1, RdRp, CP, CP-RTD, MP, and RTD was performed by using four distinct methods including single-likelihood ancestor counting (SLAC), partitioning for robust inference of selection, fixed-effects likelihood and random-effects likelihood (Scheffler et al., 2006). All these methods were employed in the adaptive evolutionary tool “Datamonkey” available online at www.datamonkey.org (Pond and Frost, 2005). To exclude the possibility of misleading results, the recombination breakpoints among all PLRV sequences (P0, P1, RdRp, CP, CP-RTD, MP, and RTD) were searched by the implementation of the Genetic Algorithm Recombination Detection (GARD) method (Kosakovsky Pond et al., 2006). Further, to analyze the mode and strength of natural selection pressure acting on the coding sequences of P0, P1, RdRp, CP, CP-RTD, MP, and RTD, the ratio of non-synonymous to synonymous substitutions (dN/dS) was estimated by SLAC method based on the GARD-corrected phylogenetic trees.

### Prediction of intrinsically disordered proteins

Several studies have been performed to predict and validate the disordered protein regions in the proteins of plant-infecting RNA viruses (Charon et al., 2016, 2018; Walter et al., 2019; Byrne et al., 2021; LaTourrette et al., 2021). In our study, the probability of protein disorder was predicted for P0, P1, RdRp, CP, CP-RTD, MP, and RTD proteins using the Protein DisOrder prediction System (PrDOS; Ishida and Kinoshita, 2007). PrDOS combines the disorder of homologous proteins and template and predicts residue disorder by a sliding window analysis of the target protein sequence. The designated PLRV reference genome sequence (NC_001747.1) was used for this purpose. The ordered and disordered protein regions were mapped and distinguished by different colors along with the prediction of the disorder probabilities. A default (0.5) threshold value indicating a false positive (FP) rate of 5% was used.

## Results

### Geographical prevalence and evolutionary relationships among PLRV isolates

**Figure 2:**
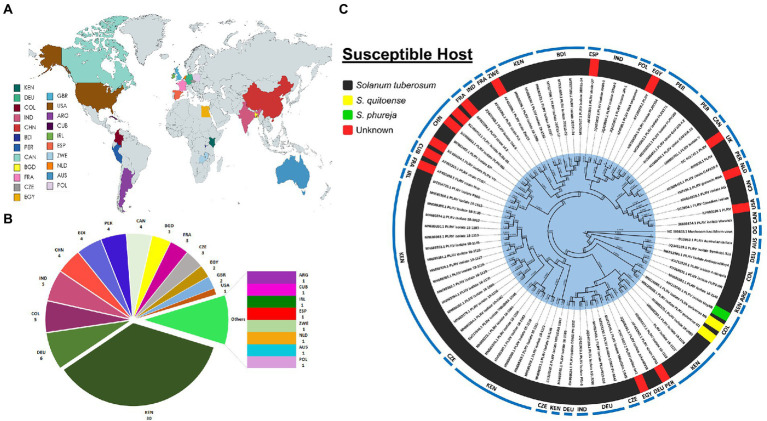
**(A)** Map indicating 22 countries with documented full-genomes of PLRV isolates. Abbreviations include KEN: Kenya, COL: Colombia, CHN: China, IND: India, CZE: Czech Republic, BDI: Burundi, DEU: Germany, PER: Peru, BGD: Bangladesh, FRA: France, EGY: Egypt, CAN: Canada, United States: United States, GBR: United Kingdom, ARG: Argentina, CUB: Cuba, IRL: Ireland, ESP: Spain, POL: Poland, NLD: Netherlands, AUS: Australia, ZWE: Zimbabwe and OG: Outgroup; **(B)** Pie chart showing the number of PLRV isolates reported from each country; **(C)** Phylogenetic tree was inferred from 84 full-genome PLRV sequences to investigate their evolutionary relatedness. The nucleotide alignments and construction of the tree were performed using MEGA-X. Bootstrap values indicate that the pattern of branching is supported by 1,000 replicates.

To date, among a total of 84 full-length globally recognized PLRV isolates, 30 isolates have been reported from Kenya, followed by Germany (6), Colombia (5), India (5), China (4), Burundi (4), Peru (4), Canada (4), Bangladesh (3), France (3), Czech Republic (3), Egypt (2), United Kingdom (2), United States of America (1), Argentina (1), Cuba (1), Ireland (1), Spain (1), Australia (1), Netherlands (1), Poland (1) and Zimbabwe (1) (Figures 2A,B). Next, to determine the standing evolutionary relatedness across all PLRV isolates, molecular phylogenetic analysis was performed. The full-length genomic nucleotide-based phylogenetic tree was created using 84 PLRV isolates reported from 22 countries (Supplementary Table S1). The majority of them originated from Kenya, infecting *S. tuberosum*. The phylogenetic analysis revealed that PLRV genomes reported from Colombia exhibited diversity regarding their associated susceptible hosts. Among three isolates that were grouped, two isolates (MN125065.1 and MN125059.1) were associated with *S. quitoense*, while one isolate (MK116549.1) was reported to infect *S. phureja* (Figure 2C). Additionally, there were 14 isolates with missing information regarding the host plants. Of 30 PLRV isolates reported from Kenya, 21 isolates formed a distinct monophyletic group. Interestingly, two PLRV isolates (EU717546.1 and EU313202.2) from the Czech Republic were also included in this clade. Further, all PLRV isolates originating from China shared genetically distinct clades with isolates reported from France, Cuba, and Ireland. The PLRV isolates originating from India shared clades with those reported from Germany and France indicating their evolutionary relatedness (Figure 2C). The nucleotide similarity index between PLRV isolates ranged between 92.5 and 100% with the lowest similarity (92.5%) observed between D13953.1 and AF453392.1 isolates originating from Australia and Peru, respectively, although the average percentage identity of aligned sequences of all these isolates was >97.7% (Supplementary Figure S2; Supplementary Table S2).

### Analysis of genetic diversity among PLRV populations

**Figure 3:**
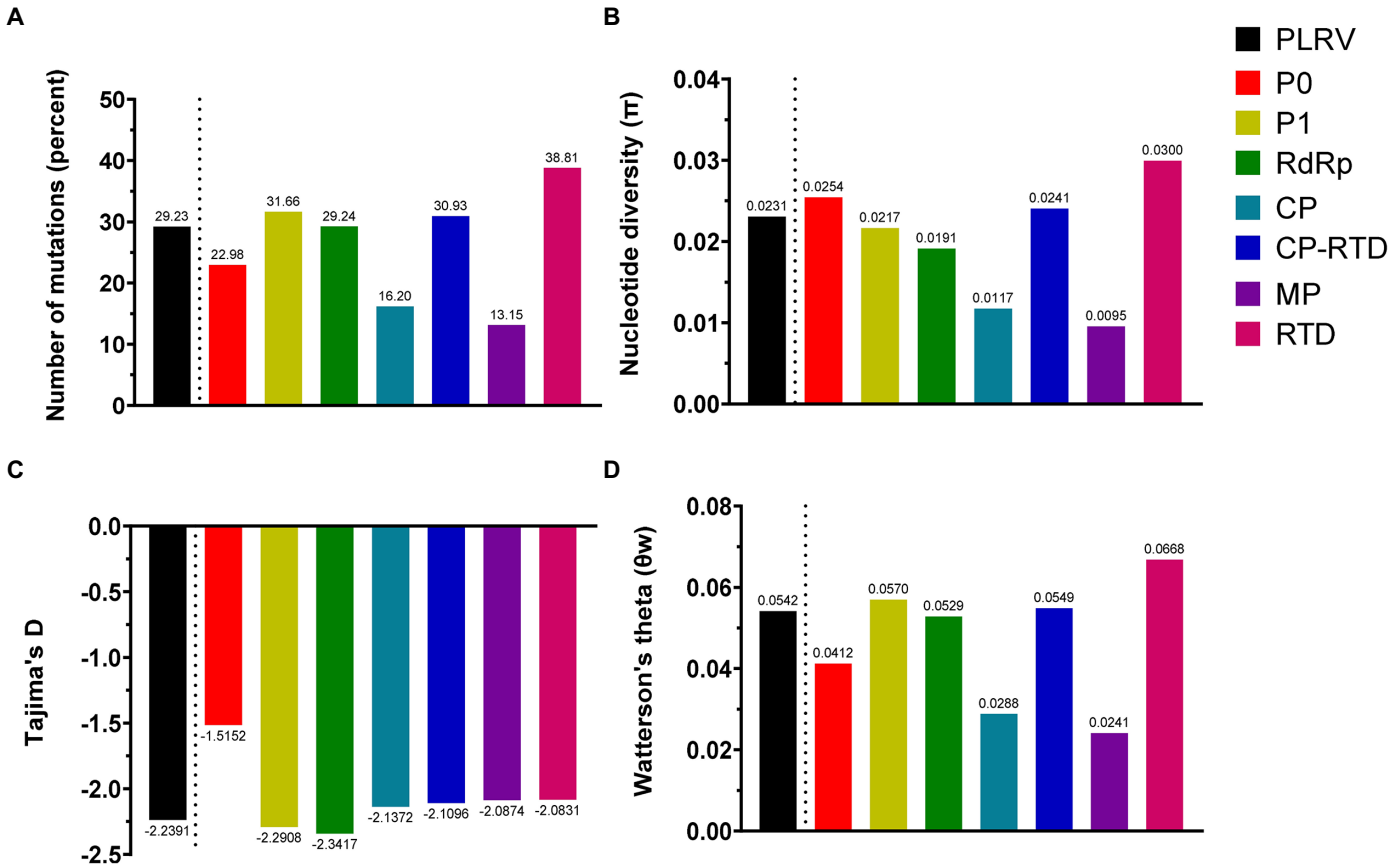
The parameters associated with genetic diversity were calculated for PLRV proteins (P0, P1, RdRp, CP, CP-RTD, MP, and RTD) using DnaSP V.5. These parameters include **(A)** number of mutations; **(B)** nucleotide diversity (π); **(C)** Tajima’s D and **(D)** Watterson’s theta (θw).

To comprehend the exact pattern of virus evolution, the comparison of molecular genetic variability among viral populations is mandatory. Therefore, we conducted an in-depth analysis of genetic variability based on all datasets containing 84 isolates of PLRV genes (P0, P1, RdRp, CP, CP-RTD, MP, and RTD). The genetic diversity, determined for the complete genome analysis of PLRV populations, revealed that PLRV had a high number of mutations (Eta = 29.23%) with a high haplotype diversity (*H_d_* = 0.999), an average nucleotide diversity (*π* = 0.02307), significantly high negative Tajima’s D value (−2.23905^**^), and an average number of segregating/polymorphic sites (*θw* = 0.05416; Figure 3; Table 1).

**Table 1:** Analysis of molecular diversity among full genomes and dynamically functional genes of PLRV global isolates.

Dataset	ORF	No. of sequences	No. of analyzed sites	*S*	*H*	*H_d_*	*π*	*θw*		Eta	Neutrality tests		
								Per site	Per sequence		Tajima’s D	Fu & Li’s D	Fu & Li’s F
PLRV	–	84	5,733	1,564	80	0.999	0.02307	0.05416	317.97386	1,676	−2.23905^**^	−3.83671^**^	−3.78200^**^
P0	P0	84	731	151	76	0.998	0.02543	0.04124	30.18751	168	−1.51524^NS^	−3.81178^**^	−3.42221^**^
P1	P1	84	1838	524	78	0.998	0.02166	0.05697	104.76294	582	−2.29075^**^	−4.32812^**^	−4.15147^**^
P1–P2	RdRp	84	3,101	820	80	0.999	0.01912	0.05285	163.93846	907	−2.34169^*^	−4.29898^**^	−4.15429^**^
P3	CP	84	617	89	64	0.990	0.01173	0.02884	17.79264	100	−2.13716^*^	−3.90396^**^	−3.81299^**^
P3–P5	CP-RTD	84	2,124	583	79	0.999	0.02407	0.05487	116.55179	657	−2.10956^*^	−3.08402^*^	−3.20218^**^
P4	MP	84	456	55	51	0.962	0.00954	0.02411	10.99545	60	−2.08740^*^	−3.94768^**^	−3.83908^**^
P5	RTD	84	1,412	472	79	0.999	0.02995	0.06683	94.36097	534	−2.08312^**^	−2.82507^*^	−3.01130^*^

S, number of segregating (polymorphic) sites; H, number of haplotypes; Eta, the total number of mutations; H_d_, haplotype diversity; θw, Watterson’s theta and π, nucleotide diversity.

* Statistically significant (*p* ≤ 0.05),

** Statistically significant (*p* ≤ 0.02), and

NS Statistically non-significant (*p* > 0.10).

The genetic diversity analysis of individual genes demonstrated that all genes were genetically variable with high numbers of polymorphisms and polymorphic sites and very high haplotype diversity as well as nucleotide diversity. Interestingly, the average number of mutations (Eta) was higher for RTD gene (38.81%), followed by P1 (31.66%), CP-RTD (30.93%), RdRp (29.24%), P0 (22.98%), CP (16.20%), and MP (13.15%; Figure 3A; Table 1). Similarly, RTD gene had the highest average pairwise nucleotide diversity (*π* = 0.0300), followed by P0 (*π* = 0.0254), CP-RTD (*π* = 0.0241), P1 (*π* = 0.0217), RdRp (*π* = 0.0191), CP (*π* = 0.0117), and MP (*π* = 0.0095), respectively. The calculated average pairwise nucleotide diversity for the PLRV population was *π* = 0.0231 (Figure 3B; Table 1).

Furthermore, we performed neutrality tests, such as Tajima’s D and Fu and Li’s D or Fu and Li’s F. Fu and Li’s D and Fu and Li’s *F* values were significantly negative for the whole genome of PLRV and *RdRP* than other genes, such as *P0*, *P1*, *CP-RTD*, *MP*, and *RTD* (Table 1). Tajima’s D value was also highly negative for *RdRP* (−2.34169), indicating the presence of polymorphic sites among this gene. Likewise, Tajima’s D value was highly negative in *P1* (−2.29075) as compared to *CP-RTD* (−2.10956) and *RTD* (−2.08312). Finally, Tajima’s D values remained highly negative for *CP* (−2.13716) followed by a closer value in *MP* (−2.08740) trailed by *P0* (−1.51524), whereas the PLRV population had highly negative Tajima’s D value (−2.23905), showing the existence of extensive polymorphic sites in the PLRV population. These observations statistically remained significant, except for the observation of *P0* (Figure 3C; Table 1).

In addition, the average number of segregating/polymorphic sites (*θw*) was higher in *RTD* gene (*θw* = 0.0668) and *P1* (*θw* = 0.0570) as compared to *CP-RTD* (*θw* = 0.0549), *RdRP* (*θw* = 0.0529), and *P0* (*θw* = 0.0412). However, the lowest values were found in the *CP* (*θw* = 0.0288) and *MP* (*θw* = 0.0241), while the value of Watterson’s theta for the full-length PLRV sequences was 0.0542 (Figure 3D; Table 1). These findings indicate that each gene has high genetic diversity displaying a high proportion of haplotypes, although *P0* and *CP-RTD* appeared to be the most genetically diverse genes in the genome of PLRV.

### Recombination is predominantly driving the genomic diversity of PLRV

**Figure 4:**
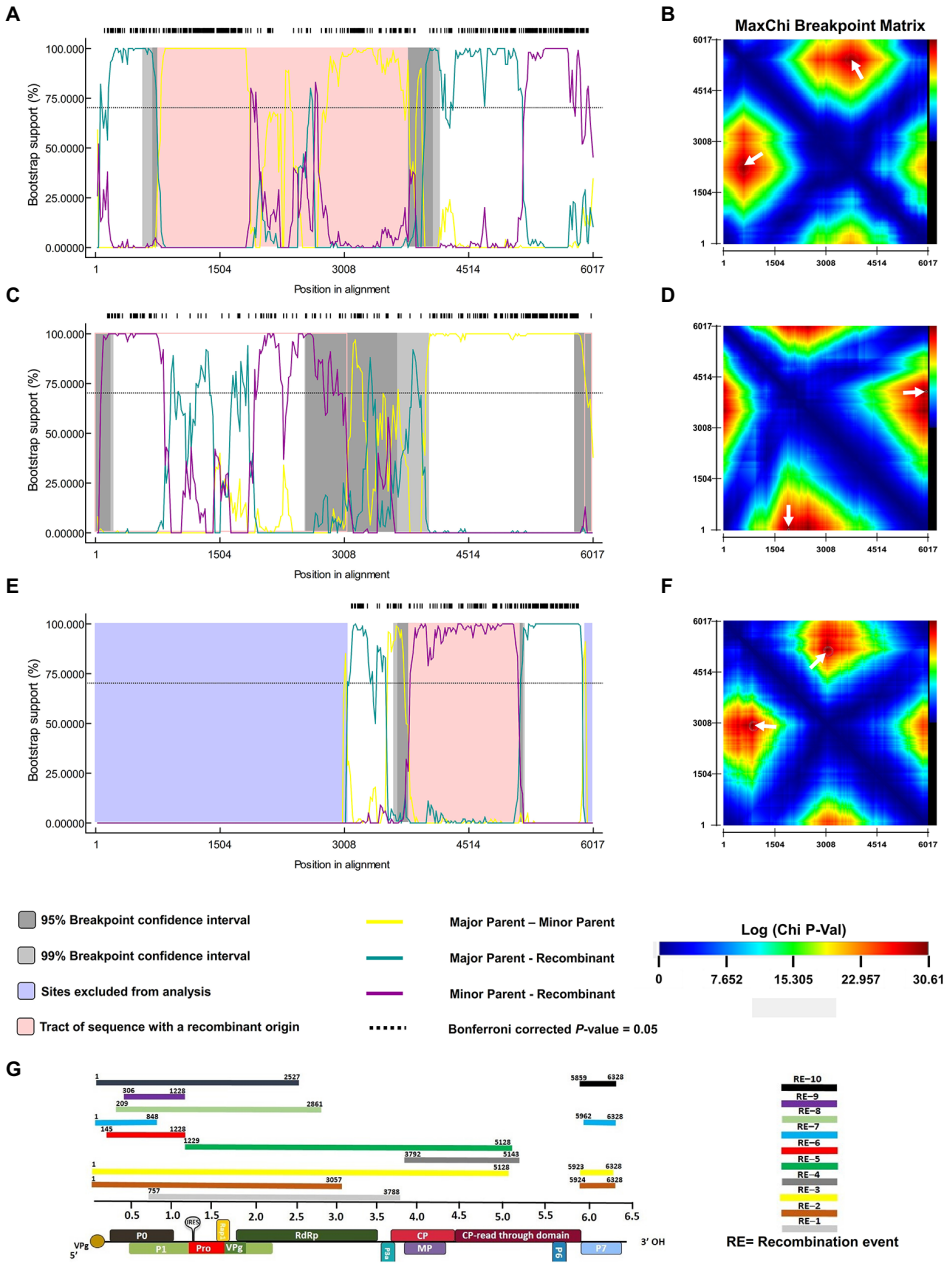
The most frequent recombination events in the PLRV genome (indicated by pink outline) were detected by using RDP V.4. Panels **(A,C,E)** represent the recombination events detected among 14, 8, and 2 isolates, respectively. The x-axis indicates the position in alignment while the y-axis denotes the percentage value for bootstrap support. Panels **(B,D,F)** represent the probabilities of recombination breakpoints best supported by *p*-values (displayed by the color key). Dark red peaks marked with white arrows indicate the statistically optimal position of recombination breakpoint pairs. Pannel G is the schematic presentation of all significantly supported recombination events found with their corresponding position on the PLRV genome.

To investigate the existence of recombination events among geographically isolated PLRV populations, we employed all seven methods, including RDP, GeneConv, Bootscan, MaxChi, Chimera, SisScan, and 3SEQ (Figure 4; Table 2). For all analyzed datasets, only the exceptional recombination events detected by at least four methods supported by a *p*-value of <0.001 were considered highly significant. The results showed that 63/84 isolates had detectable recombination events. Among them, the PLRV population that originated from Kenya was more likely to be prone to recombinational changes. For instance, a frequent recombination event was detected among 31 sequences of PLRV (*p*-value = 3.326 × 10^−17^), and the predicted beginning and ending breakpoints were located at 1125–5015 nt of the genomic RNA, covering full sequences of RdRP, CP, and MP while partial sequences of P1 and RTD ORFs (Figure 4; Table 2). The major recombinant isolate (MN689367.1) associated with this event was reported from Kenya with KY856831.1 (Argentina) and NC001747.1 (United Kingdom) being major and minor parents, respectively. Furthermore, the second most frequently detected recombination event was found in 12 isolates with a high significance (*p*-value = 4.688 × 10^−14^). The recombination breakpoints were distributed between 42 and 1,125 nt that covered a full portion of ORF0, encoding partial P0 and partial sequence of P1 ORF (Figure 4; Table 2). For this event, the major recombinant sequence was MN689365.1 (Kenya) with major (MN689381.1) and minor (KX712226.1) parents from Kenya and Colombia, respectively. Likewise, a third recombination event was found in 7 isolates (*p*-value = 2.057 × 10^−04^) at 5745–2414 nt positions and covered a partial sequence of P7, 5’ UTR, P0 (full), P1 (full), and RdRp (partial). The major recombinant isolate (MN689380.1) originated from Kenya while the major (MN689369.1) and minor (MN689384.1) parental sequences were reported from Kenya (Figure 4; Table 2). Notably, of ten significantly supported recombination events, five were associated with isolated reported from Kenya, while other recombinant isolates were reported from recombinant isolates were reported from Australia (1), Germany (1), Argentina (1), Colombia (1), and China (1).

**Table 2:** Recombination events with high significance as detected by RDP in globally-reported PLRV isolates.

Recombination event	Sequences detected with recombination Event	Recombinant sequence^1^		Recombination breakpoints with (without) gaps		ORF (without) gaps	Parental sequences		Detection Methods^2^	*P*-value^3^
		Isolate	Country	Begin	End		Major	Minor		
1	1	D13953.1	Australia	757 (653)	3,788 (3674)	P0, P1, RdRP	MK613996.1	Unknown	RGBMCS**3**	2.468 × 10^−41^
2	1	JQ346189.1	Germany	5,924 (5793)	3,057 (2927)	P0, P1, RdRP	Unknown	JQ420903.1	RGBMCS**3**	8.873 × 10^−32^
3	1	KY856831.1	Argentina	5,923 (5809)	5,128 (5014)	P0, P1, RdRP, MP, CP, RTD, P6, P7	JQ420903.1	JQ346189.1	RGMCS**3**	3.024 × 10^−19^
4	1	MN689383.1	Kenya	3,792 (3680)	5,143 (5031)	MP, CP, RTD	MN689384.1	JQ346189.1	RGBMCS**3**	1.376 × 10^−18^
5	31	MN689367.1	Kenya	1,229 (1125)	5,128 (5015)	P1, RdRp, MP, CP, RTD	KY856831.1	NC001747.1	RMCS**3**	3.326 × 10^−17^
6	12	MN689365.1	Kenya	145 (42)	1,228 (1125)	P0, P1	MN689381.1	KX712226.1	RGMCS**3**	4.688 × 10^−14^
7	3	KX712226.1	Colombia	5,962 (5856)	848 (751)	P0, P1	Unknown	MN125059.1	RGMCS**3**	1.014 × 10^−10^
8	5	MF062487.1	China	209 (99)	2,861 (2742)	P0, P1, RdRP	KY856831.1	KC456053.1	RG**M**C3	1.516 × 10^−04^
9	1	MN689382.1	Kenya	306 (202)	1,228 (1124)	P0, P1	MN689381.1	JQ420905.1	MCS**3**	3.199 × 10^−03^
10	7	MN689380.1	Kenya	5,859 (5745)	2,527 (2414)	P0, P1, RdRp, RTD, P7,	MN689369.1	MN689384.1	M**C**S3	2.057 × 10^−04^

^1^R, RDP; G, GeneConv; B, Bootscan; M, MaxChi; C, CHIMERA; S, SisScan; 3, 3SEQ. ^2, 3^The described P-value corresponds to the calculated P-value for the event in question, detected by the program in bold and underlined.

Colombian populations of PLRV were also detected to have recombinational changes. One out of five sequences was found to have recombination events (*p*-value = 1.014 × 10^−10^) at 5856–751 nt positions, which partially mapped the dynamically functional P0 and P1 ORFs (Figure 4; Table 2). Moreover, one PLRV isolate (JQ346189.1) from Germany showed recombinational changes (*p*-value = 8.873 × 10^−32^), and the predicted recombination breakpoints were located at 5793–2927 nt positions; mapping approximately half of the PLRV genome and including sub-genomic RNAs (Figure 4; Table 2). The analyses of recombination events and mapping of recombination breakpoints among the PLRV analyzed sequences signify that great diversity exists among viral populations, particularly at genomic and sub-genomic RNAs encoding various functional proteins. Our results demonstrate that some of the genes have completely recombinant sequences, such as *P0*, *P1*, *RdRp*, *CP*, *MP*, and *CP–RTD*, while *RTD* and *P6,* and *P7* have partial sequences under the effect of recombination.

### Purifying selection pressure governs The evolution of PLRV

**Figure 5:**
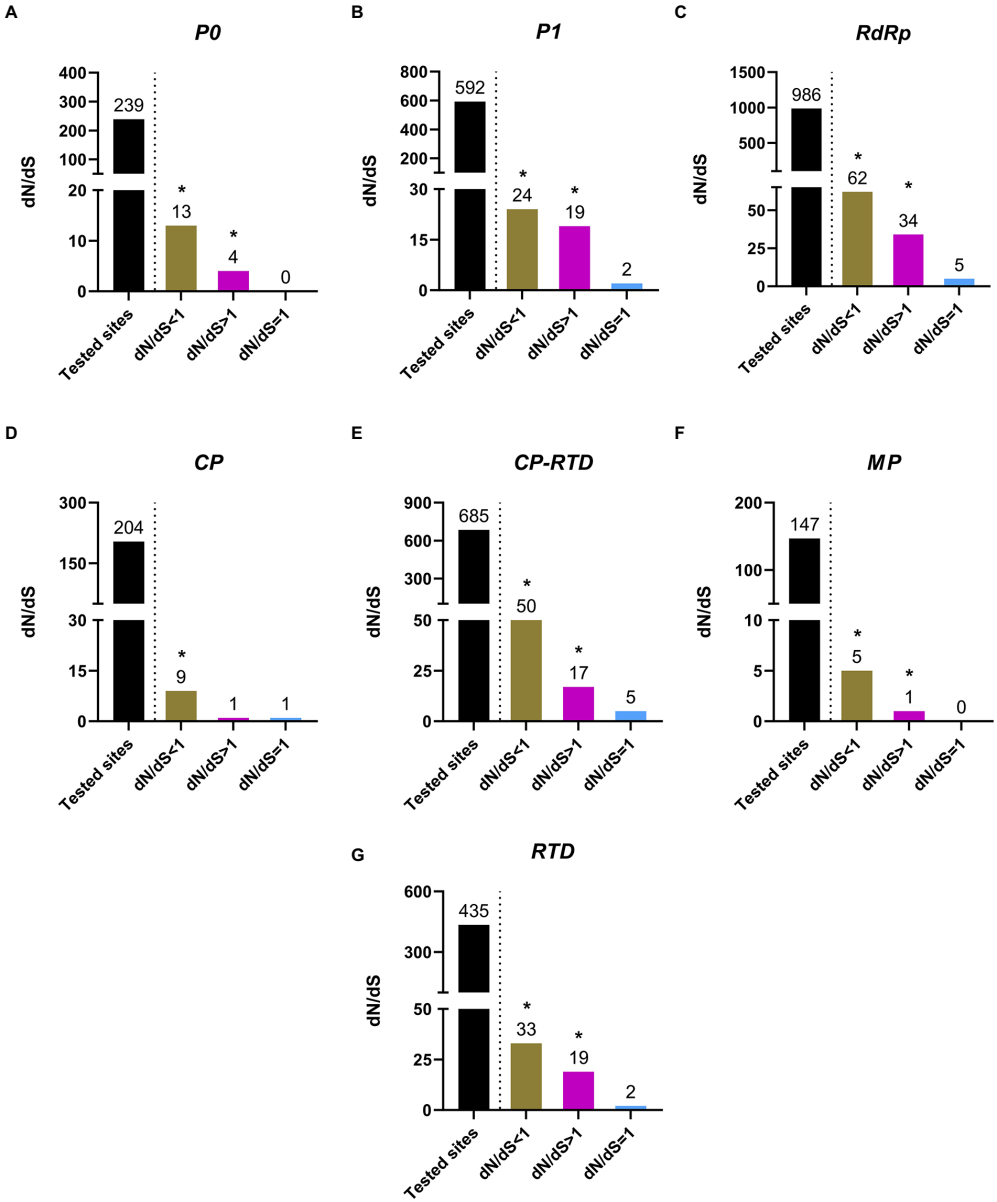
Estimation of positive (dN/dS > 1), negative (dN/dS < 1) and neutral (dN/dS = 1) selection acting upon codons of the major PLRV proteins (P0, P1, RdRp, CP, CP-RTD, MP and RTD). The * indicates that these sites were selected at the significance level of *p* < 0.1.

In order to attain a better understanding of the possible role of selection pressure in the evolution of PLRV, we compared the non-synonymous to synonymous substitutions (dN/dS) between the analyzed datasets, which included major functional proteins of PLRV, such as P0, P1, RdRp, CP, CP-RTD, MP, and RTD. Interestingly, the results indicated that the observed diverse variations on the genome of PLRV were being driven both by the positive and negative selection pressure as in addition to negative selection, several positively selected sites were also found among all PLRV genes with variable frequency (Figure 5). Particularly, both positively and negatively selected sites in the coding sequences of these genes were detected. Although most of the genes had their major part of tested sites under negative or purifying selection pressure (dN/dS < 1), the impact of positive selection (dN/dS > 1) and neutral selection (dN/dS = 1) could not be negated. However, the probability of positively and neutrally selected sites remained much lower than the negatively selected sites. Specifically, the proportion of negatively selected sites in *P0* (13/239), *P1* (24/592), *RdRp* (62/986), *CP* (9/204), *CP-RTD* (50/685), *MP* (5/147), and *RTD* (33/435) was higher compared to the positively selected sites observed in *P0* (4/239), *P1* (19/592), *RdRp* (34/986), *CP-RTD* (10/205), *CP* (1/204), *MP* (1/147), and *RTD* (19/435; Figure 5). On the other hand, P1, RdRp, CP, CP-RTD, and RTD proteins had very small and non-significant proportions of neutral selection (Figures 5B–E,G). However, P0 and MP showed no signs of neutral selection (Figures 5A,F). The considerably higher percentages of positively selected residues in RTD (4.36%) followed by RdRp (3.44%) indicate their functional importance in the relevant proteins. In conclusion, all tested proteins were observed to evolve under purifying and positive selection pressures with the former having a significant impact because the percentage of negatively selected sites was higher (Figure 5), compared with a significantly lower percentage of positive selection pressure found to variably affect all PLRV proteins (Figure 5).

### Comparison of disordered residues among PLRV proteins

**Figure 6:**
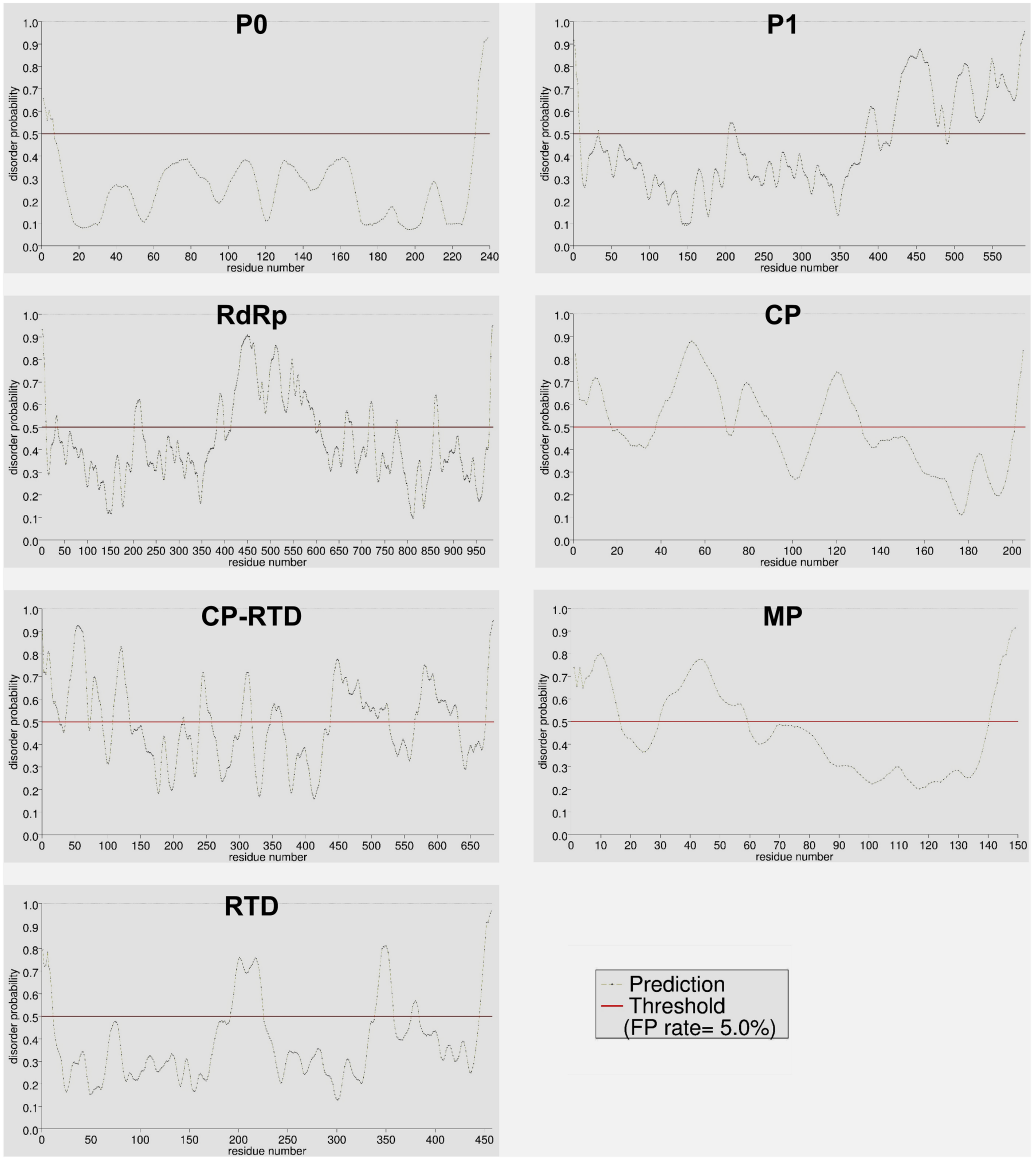
Distribution of ordered and disordered residues across different proteins (P0, P1, RdRp, CP, CP-RTD, MP, and RTD) of PLRV. Color-based coding was used to differentiate between disordered (red) and ordered (black) residues. The horizontal red line denotes a default (0.5) threshold value representing a 5% false positive (FP) rate.

**Figure 7:**
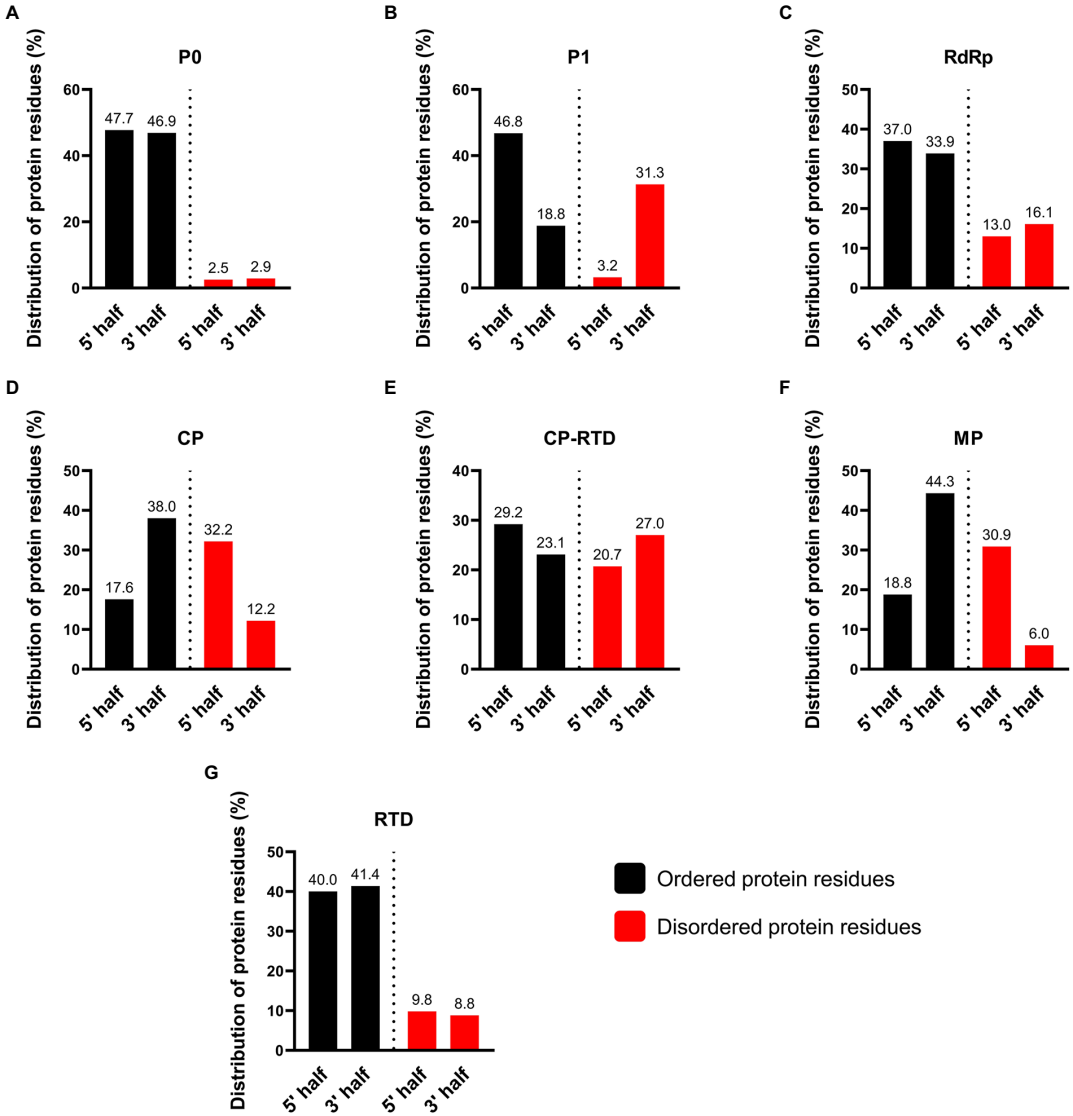
Percentage of disordered protein residues at N- and C-terminus of PLRV-encoded proteins (P0, P1, RdRp, CP, CP-RTD, MP, and RTD). Mapping of the disordered amino acids was performed using PrDOS tool. Color-based coding was used to differentiate between disordered (red) and ordered (black) residues. A default (0.5) threshold value indicating a false positive (FP) rate of 5% was used.

The intrinsically disordered proteins (IDPs) and intrinsically disordered protein regions (IDPRs) are well-known to participate in protein–protein interactions (PPIs) involving multiple or diverse binding partners. Additionally, these unstructured proteins regulate several vital processes including the assembly of protein complexes, transcription, and translation (Uversky, 2002; Szilágyi et al., 2008). The results of protein disorder prediction showed that CP-RTD contained the highest percentage (48%) of disordered amino acids followed by CP (44%), MP (37%), P1 (35%), RdRp (29%), and RTD (19%). Notably, P0 contained the lowest percentage (5.4%) of disordered residues (Supplementary Figure S1). A visual summary of the disorder probability and position in the target amino acid sequences is shown in Figure 6. We further compared the N- and C-terminals of all proteins to investigate the patterns of disordered residues. Results demonstrated that of 5.4% disordered residues of P0, approximately half (2.5%) were confined to the N-terminus, while the rest (2.9%) were detected at the C-terminus of the protein (Figure 7A). Likewise, in P1, 31.3% of disordered residues were found at the 3′ end, while only a small proportion (3.2%) of disordered amino acids was located at the 5′ half of the protein (Figure 7B). Moreover, RdRp displayed a similar pattern where 16.1% disordered residues at the 3′ end while 13% at the 5′ half were located (Figure 7C). However, CP exhibited a contrasting pattern of distribution for the disordered residues. We found that a higher percentage (32.2) of disordered residues was located at the 5′ region as compared to a lower percentage (12.2) at the 3′ end (Figure 7D). Additionally, for CP-RTD and MP proteins, the disordered residues were 20.7 and 30.9% at the 5′ half, while 27 and 6% were positioned at the 3′ end, respectively (Figures 7E,F). Finally, RTD showed a pattern similar to P0 where 9.8 and 8.8% of disordered residues were located at the 5′ and 3′ halves, respectively (Figure 7G).

## Discussion

In the present study, we performed a comprehensive analysis of the current geographical prevalence, genomic variations, recombination, and evolutionary endpoints associated with global PLRV populations. We also investigated and compared the disordered amino acids among all PLRV-encoded proteins (P0, P1, RdRp, CP, CP-RTD, MP, and RTD). We found that among full-length PLRV genomes reported from 22 countries, the PLRV isolates originating from Kenya displayed the highest genetic variability. The most significantly supported recombination event was also associated with PLRV isolates reported from Kenya. Notably, in the case of the observed type of infected host and disease prevalence, the influence of sampling biasness together with other factors cannot be ruled out (Lacroix et al., 2016). Further analysis at the individual gene level demonstrated that RTD and P1 contain the highest number of mutations. We found a considerable yet variable number of positively selected sites among all genes (*P0*, *P1*, *RdRp*, *CP*, *CP*-*RTD*, *MP,* and *RTD*). While a significant influence of purifying (negative) selection pressure seems to govern the evolution of the majority of PLRV proteins, a considerably higher percentage of positively selected amino acids in RdRp and RTD demonstrate the functional importance of these proteins. Additional results revealed that CP-RTD protein contains approximately half (48%) of amino acids identified as disordered. Given that previous studies on PLRV are either focused on one region and a single ORF (Zarghani et al., 2012) or include a few isolates (LaTourrette et al., 2021), our findings will provide detailed information on the aforementioned aspects of PLRV genetic diversity.

As mentioned earlier, PLRV belongs to poleroviruses that are exclusively vectored by aphid species (Kaplan et al., 2007). During the insect-mediated transmission and infection processes, several hosts (insects and/or plants) and environmental factors and their interactions impose certain evolutionary constraints on these viruses (Wan et al., 2015; Li et al., 2016; Nigam and Garcia-Ruiz, 2020). In the course of virus-host co-evolution, the viral and host-related factors mainly regulate the compatible or incompatible interactions (Garcia-Ruiz, 2018). The viral factors are well-known to modulate the host physiology to facilitate the dissemination of virions through insect vector feeding (Mauck et al., 2014). Additionally, climatic changes support the emergence of frequent viral epidemics by facilitating the vector populations into new and geographically isolated regions which ultimately expose new hosts to these viral pathogens (Trebicki, 2020). Thus, to reduce the chances of exclusion from the population during selection by the host (vector/plant) or the environment, viruses must attain higher levels of fitness, and maintain balanced genomic flexibility and functionality.

The genome-wide profiling of variability among P0, P1, RdRP, CP-RTD, and RTD showed that RTD has the highest percentage of mutations followed by P1, CP-RTD, and RdRP. On the contrary, the accumulation of mutations remained lowest in the ORF encoding MP. A similar trend was observed in the nucleotide diversity results where RTD displayed a higher value of Pi followed by P0 (Figure 3; Table 1). A recent study involving the combinatory analysis of PLRV and five other most variable poleroviruses demonstrates that P0 and CP-RT regions exhibited a high accumulation of nucleotide substitutions (LaTourrette et al., 2021). Further results showed that a highly negative value of Tajima’s D was associated with all genes under study, indicating the presence of excessive low-frequency polymorphism and a possible expansion in the population size after deviation from neutrality. Notably, different forms of mutations in the genomic RNA of viruses are introduced by RdRp during replication of the viral RNA (García-Arenal et al., 2001). Additionally, recombination events during the replication of viral RNA rapidly generate genetic diversity (Garcia-Ruiz et al., 2018). Both interspecific and intraspecific forms of recombination are frequently found in poleroviruses (Pagán and Holmes, 2010). The mutated, new viral genomes derived from RNA recombination might exert positive, deleterious, or neutral effects on the fitness of viruses, which consequently lead to the removal or fixation of these genomes in the viral populations (Garcia-Ruiz et al., 2018; Nigam and Garcia-Ruiz, 2020). These new genomes lead to the appearance of new strains/species of poleroviruses enabling them to cause infections in the new hosts (Ibaba et al., 2017). According to this model, it is reasonable to assume that the accumulation of mutations in the PLRV genomes is not random. Rather, preferential accumulation of mutations occurs in the proteins that are key determinants of vector transmission or host adaptation.

Recombination is the most commonly found phenomenon among +ssRNA viruses and facilitates genetic diversity *via* switching RNA genetic segments between viral isolates. This results in the introduction of new, resistance-breaking/virulent strains and host expansion (Nagy, 2008; Traoré et al., 2010; Garcia-Ruiz, 2018). In this study, we found a total of 10 recombination events spanning different regions of the PLRV genome (Table 2). The most frequently detected recombination event was associated with P0 while other recombination hotspots were also detected in other coding sequences including P1, RdRp, and CP-RTD, with variable levels of significance (Figure 4). The differential occurrence of recombination sites at the N- and/or C-terminus of these ORFs and overlapping regions suggest that possibly, different recombination-driven evolutionary histories are associated with these sites. Recent studies on poleroviruses have demonstrated that recombination breakpoints are located across the RdRp and RTD regions of *Brassica yellows virus* (BrYV; Umar et al., 2022) while recombination events in P0 and CP are hypothesized to possibly drive the evolution of *Turnip yellows virus* (TuYV; Umar et al., 2022). Likewise, studies have demonstrated that recombination events are associated with specific hotspots in the P2 and CP regions (Dombrovsky et al., 2013; Kwak et al., 2018). In the future, it will be worth studying how recombination-driven changes modulate the biological functions of these proteins.

RTD had the highest percentage of sites under negative selection followed by CP-RTD and RdRp, as compared to other genes. The highest number of positively selected sites were associated with RTD, followed by RdRp and P1. Noticeably, P0 and MP did not contain sites evolving under neutral selection pressure. Although, the ratios of negative selection associated with each ORF were variable, and there was a considerably high number of positively selected codons as well; it is evident that overall, purifying selection is the major evolutionary pressure acting on the PLRV genome (Figure 5). Likewise, findings from polerovirus-based studies (LaTourrette et al., 2021; Umar et al., 2022) suggest that the negative selection pressure acting on different ORFs of the viral genome might be essential to maintain the protein functionality, ultimately affecting the overall viral fitness.

The disordered protein regions, often referred to as IDPRs or IDPs lack a stable tertiary or three-dimensional structure and proper folding. Owing to their greater plasticity and flexibility, the IDPRs and IDPs are known to play vital roles in various biological functions (Wright and Dyson, 1999; Uversky, 2019). Some of the important biological functions associated with IDPRs and IDPs are signaling, cell regulation, survival, differentiation, proliferation, and apoptosis (Kozlowski and Bujnicki, 2012; Katuwawala et al., 2019). While some of these proteins are assumed to participate in disease etiology and possibly represent novel drug targets (Hu et al., 2016). In viruses, IDPs are recognized to govern numerous functions including adaptation to the dynamic host-related environment, counteracting host-mediated defense mechanisms, and regulation of gene expression to facilitate viral replication in the host (Gitlin et al., 2014; Xue et al., 2014; Mishra et al., 2020). In the present study, we found that CP-RTD of the PLRV contains a high percentage (48%) of disordered amino acids (Figure 7). The identified disordered regions in CP-RTD encompass domains that essentially contribute to the virion formation, systemic movement of the virus, and aphid-mediated viral transmission (Peter et al., 2008). The presence of disordered regions in the CP-RTD of PLRV elaborates the effect of host-dependent mutations in this region (Peter et al., 2008) and it might also explain that why the viral genome is capable of tolerating high rates of mutation (Tokuriki et al., 2009; Sanjuán et al., 2010). Additional research is imperative to deeply understand the correlation of high mutation rates, accumulation of disordered regions, and rapid evolution of the viral genome.

The dynamic involvement of IDPs in the protein–protein interactions could lead to the expansion of the virus host range (Charon et al., 2018). Researchers have demonstrated that in the PVY genome, IDPs might be associated with virus adaptation either by reducing the fitness cost caused by resistance-breaking mutations or by larger exploration of the evolutionary pathways. Eventually, IDPs positively affect the adaptive capacity of RNA viruses (Charon et al., 2018). From an evolutionary point of view, intrinsically disordered regions (IDRs) are thought to have a higher mutational permissiveness than highly ordered regions (Lafforgue et al., 2022). Thus, IDRs-associated amino acid polymorphism could result in the emergence of adaptive solutions during the selection process (Lafforgue et al., 2022). It has been documented that during the evolution of potyviruses, the IDPRs tend to evolve faster than the ordered regions (Charon et al., 2016). Notably, another study involving an insect-infecting RNA virus (*Nodamura virus*, NoV) demonstrates that rapidly evolving IDRs might act as the reservoir for evolutionary innovation and play vital roles in virus adaptation to new environments (Gitlin et al., 2014). In this context, the IDPs/IDPRs are hypothesized to regulate the adaptive ability of PLRV either by introducing distinct evolutionary pathways or by minimizing the mutation-induced fitness penalty; however, further experiments are imperative to validate this hypothesis.

## Conclusion

Our findings provide compelling evidence that global PLRV populations have high genetic diversity and the PLRV-encoded proteins are evolving both under positive and purifying selection with later having a more significant effect. The genome-wide profiling of variability shows that high mutation and recombination are the main factors governing the rapid evolution of PLRV genomes. The presence of a significantly high number of disordered sites in the CP-RTD region might enable PLRV to attain efficient virion formation, systemic movement, and transmission by aphid vector. Previously less-known, these mechanisms presumably are the major determinants of PLRV adaptation to new environments by broadening the host range and pathogenicity levels. These results lay solid foundations for the planning and implementation of strategies aimed at the timely diagnosis and sustainable management of PLRV.

## Data availability statement

Publicly available datasets were analyzed in this study. The names of the repository/repositories and accession number(s) can be found in the article/Supplementary material.

## Author contributions

TF and MH conceived the idea, acquired and analyzed data, and prepared the original draft. MS, HR, UW, MS, and IS analyzed data. MA, XS, and YT contributed to review and editing of the manuscript. ZH edited the manuscript, acquired funding, and supervised the study. All authors contributed to the article, and read and approved the submitted version.

## Funding

This work was funded by Discipline Team Building Projects of Guangdong Academy of Agricultural Sciences in the 14th Five-Year Period (202105TD), the President Foundation of Guangdong Academy of Agricultural Sciences, China (grant no: BZ202005), and the Project of Collaborative Innovation Center of GDAAS-XT202210.

## Conflict of interest

The authors declare that the research was conducted in the absence of any commercial or financial relationships that could be construed as a potential conflict of interest.

## Publisher’s note

All claims expressed in this article are solely those of the authors and do not necessarily represent those of their affiliated organizations, or those of the publisher, the editors and the reviewers. Any product that may be evaluated in this article, or claim that may be made by its manufacturer, is not guaranteed or endorsed by the publisher.

## Footnotes

^1^ www.ncbi.nlm.nih.gov
