# Supplementary material for: Global genetic diversity and evolutionary patterns among Potato leafroll virus populations
Source: Front Microbiol. 2022 Sep 26;13:1022016. doi: 10.3389/fmicb.2022.1022016 (PMC9801716; doi:10.3389/fmicb.2022.1022016)
Supplement: Supplementary file 2 [file Data_Sheet_2.docx]

Supplementary Material


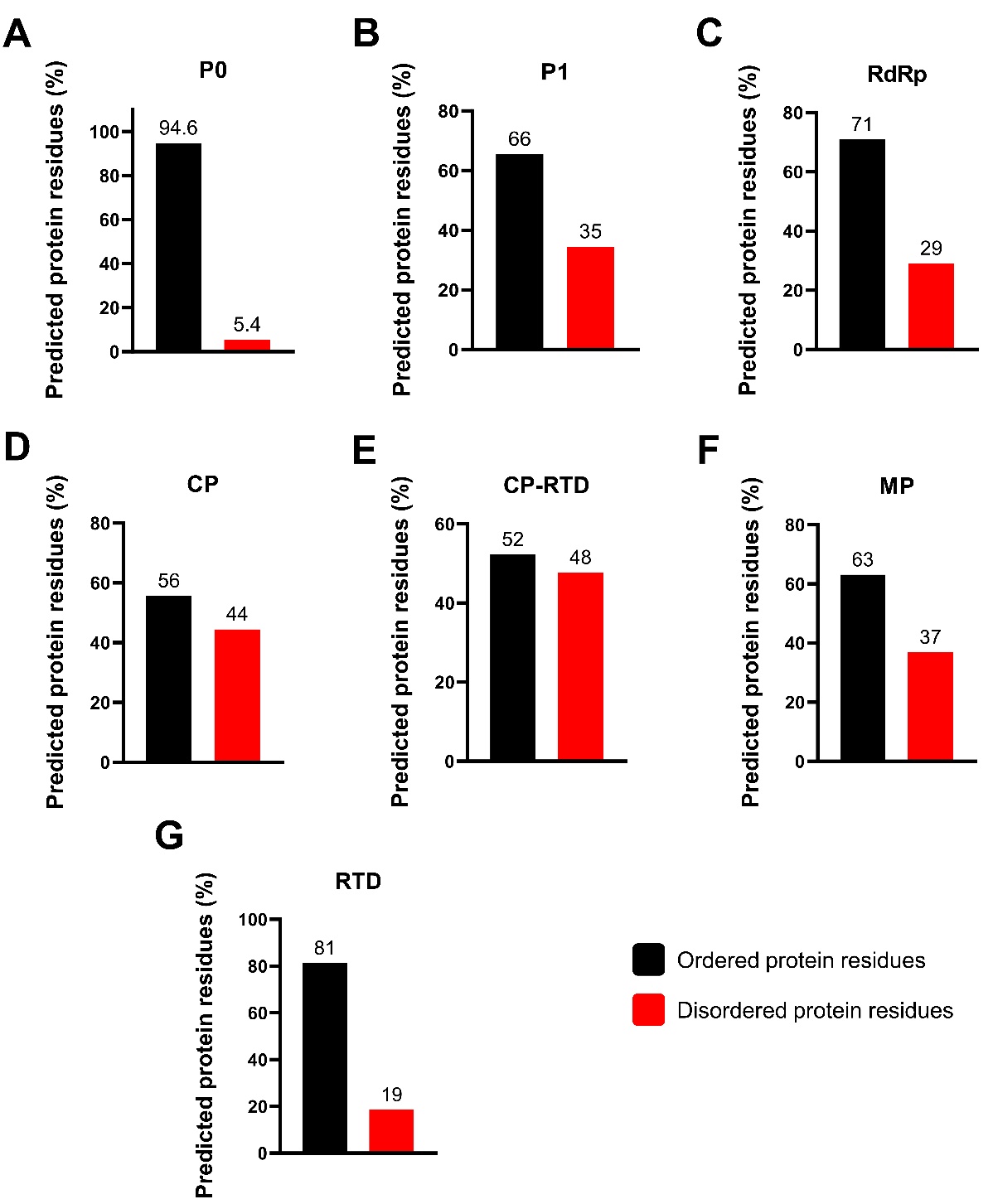


**Supplementary Figure S1.** Percentage of disordered residues associated with major proteins (P0, P1, RdRp, CP, CP-RTD, MP and RTD) of PLRV. Mapping of the disordered amino acids was performed using PrDOS tool. Color-based coding was used to differentiate between disordered (red) and ordered (black) residues. A default (0.5) threshold value indicating a false positive (FP) rate of 5% was used.


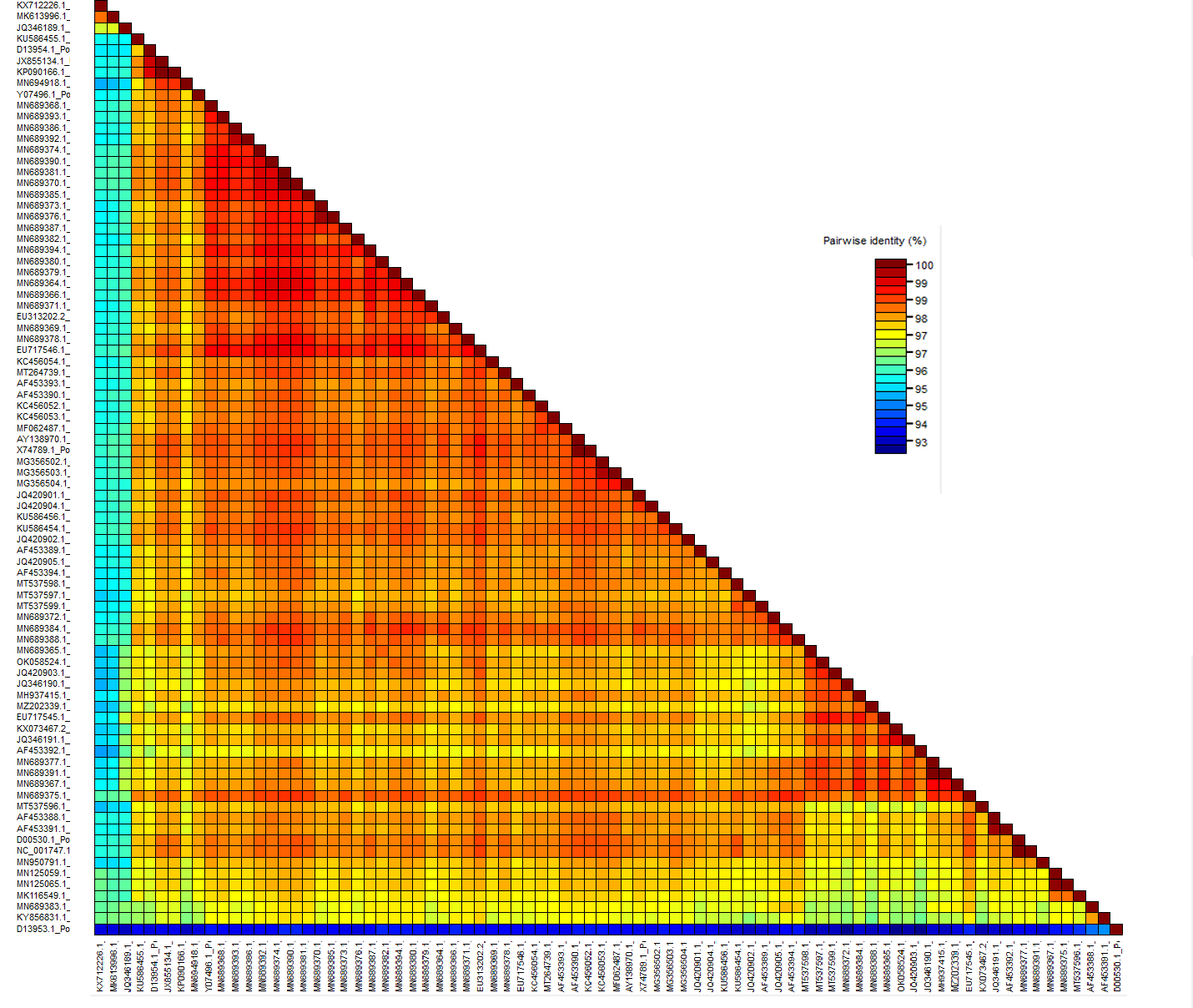


**Supplementary Figure S2.** A colored pairwise sequence identity matrix inferred using SDT v1.2 (1) showing the similarity among 84 PLRV genomes used in this study.

**Supplementary Table S1:** Description of 84 PLRV reference isolates used in this study.

| **No.** | **Accession** | **Isolate description** | **Sequence length (bp)** | **GC (%)** | **Country** | **Host** |
| --- | --- | --- | --- | --- | --- | --- |
|  | AY138970.1 | PLRV | 5884 | 49.50 | Egypt | Unknown |
|  | KP090166.1 | PLRV | 5883 | 49.50 | USA | Unknown |
|  | NC_001747.1 | PLRV | 5987 | 49.50 | UK | Unknown |
|  | MN689364.1 | PLRV isolate 18-1082 | 5886 | 49.60 | Kenya | *Solanum tuberosum* |
|  | MN689387.1 | PLRV isolate 18-1083 | 5982 | 49.40 | Kenya | *S. tuberosum* |
|  | MN689373.1 | PLRV isolate 18-1084 | 5985 | 49.30 | Kenya | *S. tuberosum* |
|  | MN689388.1 | PLRV isolate 18-1085 | 5831 | 49.90 | Kenya | *S. tuberosum* |
|  | MN689365.1 | PLRV isolate 18-1089 | 5890 | 49.40 | Kenya | *S. tuberosum* |
|  | MN689374.1 | PLRV isolate 18-1092 | 5885 | 49.70 | Kenya | *S. tuberosum* |
|  | MN689366.1 | PLRV isolate 18-1094 | 5892 | 49.50 | Kenya | *S. tuberosum* |
|  | MN689375.1 | PLRV isolate 18-1095 | 5868 | 49.60 | Kenya | *S. tuberosum* |
|  | MN689390.1 | PLRV isolate 18-1103 | 5884 | 49.80 | Kenya | *S. tuberosum* |
|  | MN689376.1 | PLRV isolate 18-1106 | 5883 | 49.50 | Kenya | *S. tuberosum* |
|  | MN689367.1 | PLRV isolate 18-1108 | 5884 | 49.70 | Kenya | *S. tuberosum* |
|  | MN689368.1 | PLRV isolate 18-1110 | 5986 | 49.60 | Kenya | *S. tuberosum* |
|  | MN689369.1 | PLRV isolate 18-1111 | 5942 | 49.70 | Kenya | *S. tuberosum* |
|  | MN689377.1 | PLRV isolate 18-1112 | 5889 | 49.40 | Kenya | *S. tuberosum* |
|  | MN689378.1 | PLRV isolate 18-1113 | 5872 | 49.60 | Kenya | *S. tuberosum* |
|  | MN689379.1 | PLRV isolate 18-1116 | 5888 | 49.70 | Kenya | *S. tuberosum* |
|  | MN689380.1 | PLRV isolate 18-1120 | 5892 | 49.40 | Kenya | *S. tuberosum* |
|  | MN689381.1 | PLRV isolate 18-1123 | 5884 | 49.70 | Kenya | *S. tuberosum* |
|  | MN689370.1 | PLRV isolate 18-1127 | 5868 | 49.70 | Kenya | *S. tuberosum* |
|  | MN689382.1 | PLRV isolate 18-1129 | 5893 | 49.60 | Kenya | *S. tuberosum* |
|  | MN689392.1 | PLRV isolate 18-1130 | 5839 | 49.60 | Kenya | *S. tuberosum* |
|  | MN689383.1 | PLRV isolate 18-1142 | 5886 | 49.50 | Kenya | *S. tuberosum* |
|  | MN689393.1 | PLRV isolate 18-1145 | 5986 | 49.60 | Kenya | *S. tuberosum* |
|  | MN689371.1 | PLRV isolate 18-1146 | 5888 | 49.60 | Kenya | *S. tuberosum* |
|  | MN689394.1 | PLRV isolate 18-1150 | 5872 | 49.70 | Kenya | *S. tuberosum* |
|  | MN689384.1 | PLRV isolate 18-1152 | 5891 | 49.50 | Kenya | *S. tuberosum* |
|  | MN689385.1 | PLRV isolate 18-1155 | 5878 | 49.60 | Kenya | *S. tuberosum* |
|  | MN689372.1 | PLRV isolate 18-1157 | 5986 | 49.40 | Kenya | *S. tuberosum* |
|  | MN689386.1 | PLRV isolate 18-1163 | 5886 | 49.60 | Kenya | *S. tuberosum* |
|  | MN694918.1 | PLRV isolate AG | 5848 | 49.70 | Canada | *S. tuberosum* |
|  | KX712226.1 | PLRV isolate Antioquia | 5881 | 49.80 | Colombia | *S. tuberosum* |
|  | MK613996.1 | PLRV isolate Antioquia/May4 | 5878 | 49.70 | Colombia | *S. tuberosum* |
|  | JQ346190.1 | PLRV isolate ASL2000 | 5865 | 49.60 | Germany | *S. tuberosum* |
|  | MF062487.1 | PLRV isolate EP | 5837 | 49.50 | China | *S. tuberosum* |
|  | KX073467.2 | PLRV isolate fer1 | 5877 | 49.40 | Egypt | *S. tuberosum* |
|  | MT537597.1 | PLRV isolate JEO11-14 | 5883 | 49.40 | Burundi | *S. tuberosum* |
|  | MT537596.1 | PLRV isolate JEO11-17 | 5882 | 49.60 | Burundi | *S. tuberosum* |
|  | MT537598.1 | PLRV isolate JEO11-22 | 5988 | 49.60 | Burundi | *S. tuberosum* |
|  | MT537599.1 | PLRV isolate JEO11-34 | 5883 | 49.70 | Burundi | *S. tuberosum* |
|  | JQ346191.1 | PLRV isolate JokerMV10 | 5865 | 49.50 | Germany | *S. tuberosum* |
|  | MK116549.1 | PLRV isolate May8B | 5850 | 49.80 | Colombia | *S. phureja* |
|  | MT264739.1 | PLRV isolate P166 | 5884 | 49.60 | Ireland | *S. tuberosum* |
|  | MG356502.1 | PLRV isolate PLRV165 | 5847 | 49.70 | Bangladesh | *S. tuberosum* |
|  | MG356503.1 | PLRV isolate PLRV171 | 5788 | 49.80 | Bangladesh | *S. tuberosum* |
|  | MG356504.1 | PLRV isolate PLRV184 | 5847 | 49.60 | Bangladesh | *S. tuberosum* |
|  | MN125059.1 | PLRV isolate PLRV_quitoense_M1 | 5987 | 49.60 | Colombia | *S. quitoense* |
|  | MN125065.1 | PLRV isolate PLRV_quitoense_M6 | 5981 | 49.60 | Colombia | *S. quitoense* |
|  | KY856831.1 | PLRV isolate PLRV-AR | 5881 | 49.80 | Argentina | *S. tuberosum* |
|  | KC456053.1 | PLRV isolate PLRV-HB | 5883 | 49.70 | China | Unknown |
|  | KC456052.1 | PLRV isolate PLRV-IM | 5883 | 49.70 | China | Unknown |
|  | KC456054.1 | PLRV isolate PLRV-YN | 5883 | 49.70 | China | Unknown |
|  | MH937415.1 | PLRV isolate PLV-W13-136 | 5883 | 49.50 | Germany | *S. tuberosum* |
|  | JQ346189.1 | PLRV isolate SymlessLS10 | 5870 | 49.50 | Germany | *S. tuberosum* |
|  | MN950791.1 | PLRV isolate V | 5848 | 49.70 | Canada | *S. tuberosum* |
|  | AF453394.1 | PLRV strain 14.2 | 5865 | 49.80 | France | Unknown |
|  | AF453392.1 | PLRV strain CIP01 | 5865 | 49.40 | Peru | Unknown |
|  | AF453393.1 | PLRV strain CU87 | 5865 | 49.70 | Cuba | Unknown |
|  | AF453391.1 | PLRV strain Fr1 | 5865 | 49.60 | France | Unknown |
|  | KU586454.1 | PLRV strain GAF318-4.2 | 5883 | 49.80 | Peru | *S. tuberosum* |
|  | KU586455.1 | PLRV strain GAF318-8 | 5885 | 49.50 | Peru | *S. tuberosum* |
|  | KU586456.1 | PLRV strain GAF318-13 | 5884 | 49.70 | Peru | *S. tuberosum* |
|  | AF453390.1 | PLRV strain Noir | 5838 | 49.70 | France | Unknown |
|  | AF453389.1 | PLRV strain OP | 5865 | 49.70 | Spain | Unknown |
|  | AF453388.1 | PLRV strain Zim13 | 5865 | 49.60 | Zimbabwe | Unknown |
|  | MN689391.1 | PLRV isolate 18-1121 | 5786 | 49.70 | Kenya | *S. tuberosum* |
|  | D00530.1 | PLRV strain 1 | 5987 | 49.50 | UK | *S. tuberosum* |
|  | D13954.1 | PLRV Canadian isolate | 5883 | 49.40 | Canada | *S. tuberosum* |
|  | JQ420901.1 | PLRV isolate JPI-1 | 5883 | 49.80 | India | *S. tuberosum* |
|  | JQ420902.1 | PLRV isolate KHPI-1 | 5883 | 49.70 | India | *S. tuberosum* |
|  | JQ420903.1 | PLRV isolate PBI-6 | 5883 | 49.70 | India | *S. tuberosum* |
|  | JQ420904.1 | PLRV isolate OTNI-2 | 5883 | 49.80 | India | *S. tuberosum* |
|  | JQ420905.1 | PLRV isolate SMI-5 | 5883 | 49.60 | India | *S. tuberosum* |
|  | JX855134.1 | PLRV isolate Warwick | 5883 | 49.50 | Canada | *S. tuberosum* |
|  | MZ202339.1 | PLRV isolate DSMZ PV-0842 | 5883 | 49.60 | Germany | *S. tuberosum* |
|  | OK058524.1 | PLRV isolate DSMZ PV-1312 | 5883 | 49.30 | Germany | *S. tuberosum* |
|  | Y07496.1 | PLRV strain Wageningen | 5882 | 49.30 | Netherlands | Unknown |
|  | D13953.1 | PLRV Australian isolate | 5882 | 49.10 | Australia | *S. tuberosum* |
|  | X74789.1 | PLRV Polish isolate | 5882 | 49.60 | Poland | *S. tuberosum* |
|  | EU313202.2 | PLRV isolate VIRUBRA 1/047 | 5814 | 49.90 | Czech Republic | *S. tuberosum* |
|  | EU717545.1 | PLRV isolate VIRUBRA 1/045 | 5801 | 49.60 | Czech Republic | *S. tuberosum* |
|  | EU717546.1 | PLRV isolate VIRUBRA 1/046 | 5779 | 49.80 | Czech Republic | *S. tuberosum* |

**References:**

1. Briddon RW, Martin DP, Roumagnac P, Navas-Castillo J, Fiallo-Olivé E, Moriones E, et al. Alphasatellitidae: A New Family with Two Subfamilies for the Classification of Geminivirus- and Nanovirus-Associated Alphasatellites. *Archives of Virology* (2018) 163(9):2587-600. doi: 10.1007/s00705-018-3854-2.
